# The prevalence of nurse burnout and its association with telomere length pre and during the COVID-19 pandemic

**DOI:** 10.1371/journal.pone.0263603

**Published:** 2022-03-16

**Authors:** Holly Wei, Julia Aucoin, Gabrielle R. Kuntapay, Amber Justice, Abigail Jones, Chongben Zhang, Hudson P. Santos, Lynne A. Hall

**Affiliations:** 1 University of Louisville School of Nursing, Louisville, KY, United States of America; 2 University of North Carolina REX Healthcare, Raleigh, NC, United States of America; 3 Piedmont Athens Regional Medical Center, Athens, GA, United States of America; 4 Biobehavioral Lab, University of North Carolina Chapel Hill, NC, United States of America; 5 Biobehavioral Laboratory and Health Resilience & Omics Science (HEROS) Hub, University of North Carolina Chapel Hill, NC, United States of America; Uniformed Services University of the Health Sciences, UNITED STATES

## Abstract

**Background:**

Burnout is a work-related stress syndrome characterized by emotional exhaustion, depersonalization, and reduced personal accomplishment. Nurse burnout is related to nurses’ deteriorating mental health and poorer patient care quality and thus, is a significant concern in healthcare. The Coronavirus Disease 2019 (COVID-19) pandemic has swept the world and distressed the healthcare systems. Because of the body’s stress mechanism, it is vital to examine the current prevalence of nurse burnout and understand it at a biological level, using an epigenetic biomarker, telomere length.

**Purpose:**

To determine the prevalence of burnout among nurses in the Peri-Operative and Labor & Delivery settings pre and during the COVID-19 pandemic and to examine the effects of burnout on absolute telomere length.

**Methods:**

This is a cross-sectional study assessing the prevalence of nurses’ burnout and the relationships between nurses’ burnout and telomere length. Due to the COVID-19 pandemic, we had to stop the study during the mid of data collection. Even though the study was not designed to capture changes before and during the pandemic, we analyzed two groups’ data before and during the pandemic. The study took place in a US hospital. Nurses in the hospital’s Operating Room, Post-Anesthesia Care Unit, and Labor & Delivery Unit participated in the study. Maslach Burnout Inventory survey and nurses’ demographics were administered online. Telomere length was measured via finger-prick blood.

**Results:**

146 nurses participated in the study, with 120 participants’ blood samples collected. The high-level burnout rate was 70.5%. Correlation analysis did not reveal a direct correlation between nurse burnout and telomere length. However, in a multiple regression analysis, the final model contained the burnout subscale of emotional exhaustion, years as an RN, and work unit’s nursing care quality. There was a low degree of departure from normality of the mean absolute telomere length in the pre-pandemic group and a substantial degree of departure in the during-pandemic group.

**Conclusions:**

Nurse burnout is a prevalent phenomenon in healthcare, and this study indicates that nurses currently experience high levels of burnout. Nurses’ cellular biomarker, telomere length, is shorter in the group of nurses during the COVID-19 pandemic than before. Appropriate measures should be implemented to decrease nurses’ burnout symptoms and improve nurses’ psychological and physical health. Nurses, especially those younger than 60, report higher burnout symptoms, particularly emotional exhaustion. This study indicates the need for intervention to promote nurses’ health during the pandemic and beyond. If not appropriately managed, nurse burnout may continue to be a significant issue facing the healthcare system.

## Introduction

Nurse burnout, a significant concern in healthcare, is a work-related stress syndrome characterized by emotional exhaustion, depersonalization, and reduced personal accomplishment [1]. The phenomenon of nurse burnout was already considered a healthcare epidemic before the Coronavirus Disease 2019 (COVID-19) pandemic, and the pandemic further worsened the situation [2, 3]. The Medscape Nurse Career Satisfaction Report [4] obtained online replies from 10,424 nurses and Advanced Practice Registered Nurses in the United States and suggested a higher burnout rate than the pre-pandemic period. The American Nurses Association (ANA) conducted two surveys, the Pulse on the National Nurses COVID-19 Survey Series: Mental Health and Wellness, in 2020. The first survey was done with 10,997 nurses in the Spring of 2020, about three to four months into the pandemic [5]. The second one included 12,881 nurses and was conducted in December 2020, a year into the pandemic [6]. The surveys indicated that nurses had intensified feelings of being overwhelmed, exhausted, anxious, sad, irritable, lonely, depressed, angry, numb, guilty, and feeling like a failure [6], which are similar to burnout symptoms.

Nurse burnout is related to nurses’ deteriorating mental health, including depression, anxiety, substance abuse, suicide rate, and poorer patient care quality [7]. Based on a meta-analysis of existing studies, there is a strong association between healthcare professionals’ burnout and patient safety actions [7]. Complicating the situation is the COVID-19 pandemic, which presented unprecedented challenges to the public and increased mental health issues among healthcare professionals [8]. Little research has examined nurses’ physical health at a cellular level using an epigenetic biomarker such as telomere length.

Telomeres are DNA-protein complexes at the end of chromosomes, and they cap chromosomal ends to promote chromosomal stability and protect the regions from degradation. Telomeres shorten during cell division with aging. Accelerated telomere shortenings are associated with chronic stress. Telomeres cannot be fully replicated when cells divide, leading to telomere shortening and eventually cells’ senescence after numerous shortenings [9, 10]. When telomeres reach a critically short length, they lose their protective function, which leads to chromosomal instability and ultimately adverse health outcomes [9]. Leukocyte telomere shortening is linked to physiological and genetic mechanisms of aging, including oxidative stress, inflammation, chronic disease, cellular senescence, mortality [9, 10], and psychological illnesses, such as depression, posttraumatic stress disorder, and hostility [11]. Thus, telomere length is a measure of cumulative biological weathering and accelerated aging due to chronic stress, situating telomeres as a candidate biomarker of stress-related disorders or a stand-alone measure of age-related declines across systems [9, 10].

Because high burnout and psychological distress can accelerate molecular aging [12], it is crucial to understand whether nurses’ burnout is associated with accelerated molecular aging as measured by telomere length. The purposes of this study were to determine the prevalence of burnout among nurses pre and during the COVID-19 pandemic and to examine the effects of burnout on absolute telomere length. The overall hypothesis was that greater burnout symptoms pre and during the COVID-19 pandemic would be associated with shorter telomere length.

### Theoretical framework

The theoretical framework guiding this study is the Allostatic Load Theory [13, 14]. This framework describes the wear-and-tear process of allostasis by repeated physiological responses to psychological stress, leading to allostatic overload and eventually poor health outcomes. Longitudinal studies linked stress with disease states such as metabolic syndrome, cardiovascular disease, diabetes, and other diseases of aging [13]. The general and pervasive effects of stress are related to changes proximal to the stress response, such as changes in the regulation of the HPA axis and common cellular mechanisms. When individuals are challenged repeatedly by stressors or when the allostatic systems remain turned on when no longer needed, the mediators of allostasis can produce wear and tear on the body and brain, leading to allostatic overload [13, 14].

Burnout may be associated with sustained autonomic nervous system activation and sympathetic adrenal medullary axis dysfunction [12]. Given that telomere length is related to various stressor exposures, we conceptualize telomere length as a potential molecular-level measure of allostatic load and a proxy for the HPA axis and cellular mechanisms. Allostatic load incorporates dysregulation across multiple systems, and telomere length may index cumulative inputs from various regulatory systems.

### Hypotheses

The hypotheses were:

H_1_: Absolute telomere length is negatively related to the emotional exhaustion component of burnout.
H_2_: Absolute telomere length is negatively related to the depersonalization component of burnout.
H_3_: Absolute telomere length is positively associated with the personal accomplishment component of burnout.
H_4_: The absolute telomere length of nurses pre-pandemic is longer than that of nurses during the pandemic.
H_5_: The nurses’ burnout scales and demographic characteristics will account for a significant portion of the variance in absolute telomere length.

## Methods

### Study design

This was a cross-sectional study assessing the prevalence of nurses’ burnout and the relationships between nurses’ burnout and telomere length. Due to the COVID-19 pandemic, we had to stop the study during the mid of data collection. Even though the study was not designed to capture changes before and during the pandemic, we had two groups’ data before and during the pandemic. Thus, we also conducted a comparative analysis of nurses’ telomere length pre- and during the COVID-19 pandemic.

### Setting and sample

The study took place in a university-associated hospital on the east coast of the United States. This is a tertiary care, not-for-profit hospital with 439 in-patient hospital beds and 2000 registered nurses. Nurses in the hospital’s Operating Room, Post-Anesthesia Care Unit, and Labor & Delivery Unit were invited to participate. These units were selected because there is little research on nurses’ burnout in peri-operative units, and there was a call for proposals on peri-anesthesia nurses’ health. A power analysis was conducted using the software of G*Power 3.1 [15]. The analysis was performed for multiple linear regression with a fixed model, assuming 80% power, α = .05, and an effect size of R^2^ = .20, as recommended by Polit and Beck [16]. A total sample size of 75 was needed for the study. Inclusion criteria were: a) hospital’s registered nurses, b) 21 years of age or older, and c) work on the units for at least six months. Exclusion criteria were unwilling to answer the surveys or provide a one-time blood sample from a finger-prick for telomere length determination.

### Measures

#### Maslach burnout inventory

The Maslach Burnout Inventory-Human Services Survey for Medical Personnel is a popular scale for measuring burnout symptoms in healthcare professionals. It consists of 22 items that assess three aspects of burnout: Emotional Exhaustion (EE; 9 items), Depersonalization (DP; 5 items), and low sense of Personal Accomplishment (PA; 8 items). Each item is rated on a 7-point Likert scale of 0 = *Never*, 1 = *A few times a year or less*, 2 = *Once a month or less*, 3 = *A few times a month*, 4 = *Once a week*, 5 = *A few times a week*, or 6 = *Every day*. Cronbach’s alphas for the subscales were: Emotional Exhaustion (.92), Depersonalization (.73), and Personal Accomplishment (.68).

#### Telomere length and quantification

Nurses’ blood spot samples were collected via finger prick on Whatman paper cards (Catalog No 10534612) following kit instructions. There were two findings related to the correlation between blood spot samples and whole blood samples. In 2013, Zanet et al. [17] found that telomere length measurements were significantly higher in dried blood spots collected directly from fingertip prick than dried blood spots prepared with anticoagulated whole blood collected from the finger and non-blotted whole blood taken from both finger and arm venipuncture. In 2017, Stout et al. [18] found a significant correlation between blood spot samples and whole blood samples. In this study, genomic DNA was extracted from the blood spots with QIAamp DNA kit (Catalog No 56504) per instructions.

Telomere length quantification was obtained using ScienCell Absolute Human Telomere Length Quantification (AHTLQ) qPCR assay kit (Catalog No 8918). Specifically, the telomere primers recognize and amplify telomere sequences. The single-copy reference primers recognize and amplify a 100bp-long region on human chromosome 17, serving as a reference for data normalization. Reference genomic DNA with known telomere length was used as a reference for calculating the absolute telomere length of target samples. Two consecutive qPCR reactions were set for each target DNA sample, one to amplify the telomere sequence and the other one to amplify the SCR sequence. Both qPCR reactions were performed in triplicates with a final reaction volume of 10μL which included 2μL of target or reference genomic DNA, 1μL of primer stock solution, 5μL of 2X qPCR GoldNStart TaqGreen master mix, and 2μL of nuclease-free water. The PCR conditions were initial denaturation at 95°C for 10 min, followed by 32 cycles of 95°C for 20 s, 52°C for 20 s and 72°C for 45 s. Relative telomere length fold changes to the reference human genomic DNA were calculated using ΔΔCT method. As the telomere length of the reference genomic DNA was known, absolute human telomere length could be calculated accordingly. This study reports telomere length in the absolute length per diploid cell or per chromosome end. The absolute length is the actual length and is expressed as the kilobase (kb) value per diploid cell.

#### Nurses’ demographic characteristics

Nurses’ demographic characteristics were collected via a demographic survey designed by the researchers based on the study purpose. The information collected included nurses’ age, work experiences, years as an RN, night shift frequency, exercise frequency, intent to leave, job satisfaction (not satisfied to very high), life stress levels (no to very high), and perceptions of patient care quality (very poor to very high).

### Ethical considerations and procedures

The university institutional review board approved this study.

### Data collection

Data collection occurred in the hospital’s Operating Room, Post-Anesthesia Care Unit, and Labor & Delivery Unit. The research team recruited participants using several strategies, including posting information flyers on the unit, introducing the study at the units’ staff meetings, and sending emails to nurses. The information on the flyers and emails included the title, purpose, description of the study, eligibility to participate, and contact information. Nurses interested in knowing more about the study could contact the research team to ask questions.

REDCap, a browser-based web application for managing online surveys, was used to upload the study surveys and build an online survey link. The survey link was distributed via units’ listservs, and nurses who were interested in participating would open the link. Upon opening the link, participants were instructed to read the study description. If they had questions, they could still call the phone number in the email for more questions. After reading the study instructions and requirements, nurses could click Agree to participate or No to end. Each participant who answered the survey received a personal number at the end of the survey, which was placed on the blood sample to match the participants’ survey and blood sample results. Nurses’ paper-copy informed consents were obtained at the time of blood sample collection. Research team members collected the participants’ blood samples via finger pricks in the hospital.

### Data analysis

The IBM^®^ Statistical Package for the Social Sciences (SPSS)^®^ Statistics v. 27 was used for the data analysis. Frequency distributions were calculated for categorical variables, including demographic characteristics. Descriptive statistics such as means, medians, standard deviations, and ranges were used to summarize the continuous variables. Pearson’s product-moment correlations were calculated to evaluate the strength of relationships between the variables, including each subscale of the MBI and telomere length. Independent samples *t*-tests were run to determine if there were differences in mean burnout scale scores and absolute telomere length between nurses pre and during COVID-19. Multiple regression with a backward elimination stepwise variable retention procedure which maximizes the retention of valuable contributors to the model was used to determine if the burnout scales and demographic characteristics predicted absolute telomere length. Because age is a confounding factor in telomere studies, we controlled for age when analyzing telomere-related data, specifically when comparing telomere lengths before and during the pandemic (Tables 5 and 6) and conducting the backward elimination multiple regression (Table 7). A two-way ANOVA was conducted to examine the effect of age and the pandemic (pre and during the pandemic) on telomere lengths. All hypotheses were tested using an alpha of .05 for statistical significance.

## Results

### Demographic characteristics of the sample

A total of 146 nurses participated in the study, with 120 participants’ blood samples collected. The remaining participants could not be reached for blood collections. Thus, the effective sample size for hypothesis testing was 120.

Table 1 presents the frequency distributions for the demographic characteristics of the sample. The sample was predominantly female and Caucasian; the median age was approximately 45 years. Most of the nurses had a bachelor’s degree or higher and were at the second of the four levels of seniority. The median number of years of experience as an RN was approximately 20. Most nurses worked in either the Operating Room or the Post-Anesthesia Care Unit. Descriptive statistics for absolute telomere length and the independent variables are presented in Table 2.

**Table 1:** Frequency distributions and percentages of categorical demographic variables (n = 136).

Variable	Category	Frequency (n)	Percent (%)
Age in years	20–29	31	21.2
	30–39	28	19.2
	40–49	25	17.1
	50–59	34	23.3
	60 or above	18	12.3
Gender	Male	6	4.1
	Female	129	88.4
	Other	1	0.7
Race	American Indian or Alaska Native	1	0.7
	Asian or Asian American	7	4.8
	Black or African American	9	6.2
	Hispanics of any race	2	1.4
	Native Hawaiian or other Pacific Islander	1	0.7
	White or Caucasian	114	78.1
	Two or more races	2	1.4
Education	Diploma	5	4.0
	Associate Degree	20	15.0
	Bachelor	103	75.0
	Master	7	5.0
	Doctorate	1	0.7
Job title	RN CN I (Clinical Nurse)	8	6.0
	RN CN II (Clinical Nurse)	98	72.0
	RN CN III (Clinical Nurse)	12	9.0
	RN CN IV (Clinical Nurse)	7	5.0
	RN Managers (Supervisory)	11	8.0
Years as an RN	< 1	8	5.5
	1–4	29	19.9
	5–9	15	10.3
	10–14	12	8.2
	15–19	16	11
	20–24	7	4.8
	25–29	15	10.3
	>30	34	23.3
Work Unit	Post-Anesthesia Care Unit	41	28.1
	Operating Room	56	38.4
	Labor Delivery	23	15.8
	Mother-Baby	1	0.7
	Surgical unit	7	4.8
	Other	8	5.5

**Table 2:** Descriptive statistics for the study variables.

Variable	n	Minimum	Maximum	Mean	SD
Telomere Total Length	120	46.2	999.30	355.95	251.363
MBI-Emotional Exhaustion	146	13	60	32.39	11.603
MBI-Depersonalization	146	5	31	9.88	4.862
MBI-Personal Accomplishment	146	30	56	47.68	5.463
Exercise frequency (number of days)	136	1	8	3.17	1.689
Night shift frequency (number of days)	136	1	5	1.32	0.926
Stress perceived item	136	1	5	2.75	0.841
Life stress outside of work item	136	1	5	3.11	0.932
Burnout perceived item	136	1	5	3.17	1.086
Job satisfaction item	136	1	5	2.76	0.839
Effectiveness handling work stress item	136	1	4	1.94	0.653
Stress impact on patient care item	136	1	5	3.88	0.864
Work unit’s nursing care quality item	136	1	4	1.97	0.620

### The prevalence of nurses’ burnout

Table 3 presents that nurses’ burnout rates. A majority of the nurses experienced high emotional exhaustion and, to a lesser extent, high depersonalization. Few reported low personal accomplishments. The total high burnout rate (the number of nurses who scored higher than the cutoff on one or more subscales) was 70.5%.

**Table 3:** Prevalence of burnout among nurses in the sample (N = 146).

MBI Subscales	Burnout Levels	Frequent	Percent
Emotional Exhaustion (Score: 0–54)	High (≥ 27)	95	65.1
	Moderate (19–26)	35	24.0
	Low (0–18)	16	11.0
Depersonalization (Score: 0–30)	High (≥ 10)	56	38.4
	Moderate (6–9)	68	46.6
	Low (0–5)	22	15.1
Personal Accomplishment (Score: 0–48)	High (0–33)	1	0.7
	Moderate (34–39)	13	8.9
	Low (≥ 40)	132	90.4
Total Burnout (Total High Burnout refers to the number of nurses who scored higher than the cutoff on any subscales). The cut-off values are based on each subscale’s cut-off values listed above.	Low Burnout	43	29.5
	High Burnout	103	70.5

### Nurses’ burnout symptoms and telomere length

#### Nurses’ emotional exhaustion and telomere length

Hypothesis 1 proposed a negative relationship between the emotional exhaustion component of burnout and telomere length. This hypothesis was tested by correlating the scores on the Emotional Exhaustion scale of the MBI with the telomere length measurements using the product-moment correlation. This correlation was computed to be .133, for which the one-tailed *p*-value = .927. This *p*-value exceeds the .05 threshold for statistical significance; thus, this hypothesis was not supported (*r* = .13, *p* = .146).

#### Nurses’ depersonalization and telomere length

Hypothesis 2 proposed that there was a negative relationship between the depersonalization component of burnout and telomere length. This hypothesis was tested by correlating the scores on the Depersonalization scale of the MBI with the telomere length measurements using the product moment correlation. This correlation was computed to be .136, for which the one-tailed *p*-value = .931. This *p*-value exceeds the .05 threshold for statistical significance, and accordingly, the hypothesis was not supported (*r* = .14, *p* = .139).

#### Nurses’ personal achievement perceptions and telomere length

Hypothesis 3 proposed a positive relationship between the personal accomplishment component of burnout and telomere length. This hypothesis was tested by correlating the scores on the Personal Accomplishment scale of the MBI with the telomere length measurements using the product-moment correlation. This correlation was computed to be -.028, for which the one-tailed *p*-value = .617. This *p*-value exceeds the .05 threshold for statistical significance, and this hypothesis was not supported (*r* = -.03, *p* = .766). The data provide no evidence of associations of the dimensions of burnout with absolute telomere length.

### Nurses’ telomere length and the advent of the COVID-19 pandemic

Hypothesis 4 proposed that nurses’ mean absolute telomere length shortened after the advent of the pandemic. This hypothesis was tested by comparing the means of the telomere length measurements obtained from one group of nurses before the advent of the pandemic and a separate group of nurses during the advent of the pandemic. An assessment of the degree of departure from normality of the telomere length measurements in these two groups revealed a low degree of departure from normality in the pre-pandemic group (Shapiro-Wilk *W* = .946) but a substantial degree of departure in the during-pandemic group (Shapiro-Wilk *W* = .777). Consequently, this hypothesis was tested by applying the parametric independent samples *t*-test. The means of the two groups are presented in Table 4, followed by the results of the statistical tests in Table 5. The results of the tests of the differences between pre and during pandemic telomere lengths were significant using both the parametric and nonparametric tests (one-tailed *p* < .001 in both cases). The two-way ANOVA conducted to examine the effect of pandemic (pre and during the pandemic) on the telomere length controlling for age (Table 6) showed that: 1) There are differences in total/average telomere lengths between different age groups (*P* = 0.11); 2) There are differences in total/average telomere lengths between pre- and during-pandemic (*P* < 0.001); 3) There are no interactions between nurses’ age and their pre- and during pandemic telomere lengths (*P* = .654). Regardless of age, nurses’ pre-COVID-19 total/average telomere lengths were longer than during the COVID-19 pandemic.

**Table 4:** Descriptive statistics for telomere length measures by pre- and during pandemic groups.

Variable	Group	N	Mean	SD
Telomere Total Length	Pre-Pandemic	52	499.454	248.351
	During-Pandemic	68	246.216	192.531
Telomere Average Length	Pre-Pandemic	52	5.429	2.699
	During-Pandemic	68	2.677	2.093

**Table 5:** Results of parametric and non-parametric tests of pre- vs. during pandemic telomere lengths.

Variable	Pre & During Difference	*t*	*df* *	*p*	Mann-Whitney U	*z*	*p*
Telomere Total Length	253.238	6.086	93.60	< .001	733.5	-5.479	< .001
Telomere Average Length	2.752	6.085	93.61	< .001	734.5	-5.473	< .001

* Levine test for homoscedasticity *p* < .001, equal variances not assumed, Satterthwaite correction to degrees of freedom applied.

**Table 6:** Between-subjects effects pre- and during pandemic on telomere length controlling for age.

Source	Type III Sum of Squares	df	Mean Square	F	Sig.
Corrected Model	2614960.785^a^	9	290551.198	6.521	.000
Intercept	14440561.630	1	14440561.630	324.098	.000
Age	612236.706	4	153059.177	3.435	.011
Pre-, During-COVID	2026127.050	1	2026127.050	45.474	.000
Age * Pre-, During COVID	109413.604	4	27353.401	.614	.654
Error	4856620.158	109	44556.148		
Total	22703627.960	119			
Corrected Total	7471580.943	118			

Note: Levine test for homoscedasticity *p* < .02, equal variances not assumed.

### Nurses’ burnout symptoms, demographic items, and telomere length

Hypothesis 5 proposed that nurses’ burnout symptoms and the demographic items would explain a significant proportion of variance in telomere length. This hypothesis was tested using hierarchical multiple regression, both controlling and not controlling for age. Regardless of age, the models’ *p*-values are significant (*P* < .001). The three independent variables left in the model are MBI—Emotional Exhaustion, Years as RN, and Work unit’s nursing care quality. This study provided evidence that a subset of the salient potential predictors identified may explain a significant proportion of the variance in telomere length. The predictive variables, their coefficients, and their individual *p*-values are presented in Table 7.

**Table 7:** Results of backward elimination multiple regression of potential predictors of total telomere length (n = 120).

Model Term	Unstandardized Coefficients		*t*	*p*
	*B*	Std. Error		
(Constant)	485.037	111.071	4.367	< .001
Age	23.685	33.155	.714	.476
MBI–Emotional Exhaustion	3.911	1.996	1.959	.053
Years as RN	-37.806	18.292	-2.067	.041
Work unit’s nursing care quality	-83.272	37.209	-2.238	.027

To help understand the phenomenon, we examined the descriptive statistics for telomere length and MBI-Emotional Exhaustion scores based on nurses’ age groups (Table 8). Table 8 shows that younger nurses generally had longer telomere lengths but had higher emotional exhaustion symptoms. Nurses > 60 had the shortest telomere lengths and the lowest emotional exhaustion scores.

**Table 8:** Nurses’ age groups, telomere lengths, and emotional exhaustion.

	Telomere Length							MBI–Emotional Exhaustion			
		Telomere Total Length			Telomere Average Length						
Age Group (years)	N	Mean (Std. Deviation)	Min.	Max.	Mean (Std. Deviation)	Min.	Max.	N	Mean (Std. Deviation)	Min.	Max.
20–29	31	461.43 (262.57)	106.10	920.20	5.02 (2.85)	1.15	10.00	31	32.81 (11.56)	16	56
30–39	24	330.00 (262.45)	68.20	999.30	3.59 (2.85)	.74	10.86	28	33.93 (11.73)	16	59
40–49	22	337.08 (242.93)	51.50	791.20	3.67 (2.64)	.56	8.60	25	30.72 (10.70)	14	54
50–59	28	334.52 (239.55)	103.10	957.10	3.64 (2.60)	1.12	10.40	34	34.06 (12.99)	13	60
> 60	15	268.65 (209.53)	46.20	794.90	2.92 (2.28)	.50	8.64	18	26.33 (8.17)	13	45
Total	120	357.77 (251.63)	46.20	999.30	3.87 (2.74)	.50	10.86	136	32.11 (11.56)	13	60

## Discussion

This study explored the effects of nurses’ burnout on the cellular level using an epigenetic biomarker, absolute telomere length, during the COVID-19 pandemic. In addition, because this study crossed two time periods pre and during the COVID-19 pandemic, we compared nurses’ burnout and absolute telomere length in these two independent samples.

The study findings suggested significant differences between nurses’ telomere lengths pre and during the pandemic, with significantly shortened telomere lengths in the during-pandemic group of nurses compared with the pre-pandemic group. Knowing that age is a significant confounder, we analyzed telomere-related data controlling for age. As expected, younger nurses, age group 20–29, have the longest telomere lengths, and as ages increase, telomere length becomes shorter. However, regardless of nurses’ age group, we saw significantly shorter telomere length during the pandemic than before the pandemic in this group of nurses, suggesting the pandemic’s influence on nurses’ telomere lengths.

While we expected nurses’ burnout would have significant correlations with their telomere lengths, the relevant hypothesis was not supported. The data did not indicate significant bivariate relationships between absolute telomere length and nurses’ burnout symptoms. Findings from this study showed that telomere lengths were shorter among participants recruited during the pandemic onset as compared to those recruited pre-pandemic although no causal linkages can be made with these cross-sectional data. When we conducted multiple regression analysis, significant predictors of telomere lengths included MBI Emotional Exhaustion, years as an RN, and nurses’ perceptions of nursing care quality on their unit; these variables explained a significant portion of the variance in absolute telomere length. As a proxy for age, years as an RN were negatively associated with nurses’ telomere length.

The common belief is that psychological issues negatively correlate with telomere length, where significantly shorter telomere was identified in individuals with psychological disorders [19]. The findings, however, suggested that emotional exhaustion had a positive relationship with telomere length. The greater the emotional exhaustion reported, the longer the telomere length. In addition, nurses’ perception of the work unit’s nursing care had a negative relationship with telomere length. Thus, the lower/poorer the perception of quality nursing care, the longer the telomere length. These results are contradictory with current literature and the theoretical framework proposed. One possible explanation for this finding is that higher emotional exhaustion was reported by younger nurses in the present sample, and thus it would be expected that they would have longer telomeres.

Even though past research has recognized the potential direct relationships between nurses’ age and burnout, the research results are inconclusive [20]. Our data show that nurses in the younger age groups have higher emotional exhaustion scores (≥ 27 = cut-off values for high-level burnout) than the nurses over the age of 60 (< 27). The literature on the relationships between age and burnout indicated that older nurses or workers reported lower levels of burnout [21–24]. These research findings suggest that older nurses may have more cognitive, psychological, and professional strengths that advance with age compared to younger nurses. These reports were consistent with the current study’s finding, offering evidence that hospitals may pay attention to young and mid-age nurses concerning nurse burnout symptoms.

The allostatic load theoretical framework indicates that prolonged stress can result in allostatic load (wear-and-tear on the body), leading to impaired immunity and other diseases such as atherosclerosis, bone demineralization, and brain nerve cell atrophy [25]. These findings help us understand nurses’ burnout at the cellular level and may contribute to designing interventions to improve nurses’ psychological and physical health and prevent the earlier onset of age-related diseases. The COVID-19 pandemic induced high-stress levels in individuals at the frontline [11, 26], and stress reactivity can trigger the shortening of telomeres [27]. This study shows the necessity of a follow-up study to observe this population for potential long-term burnout and telomere effects.

Physically and emotionally exhausted nurses have increased absenteeism, turnover, and presenteeism, defined as productivity loss due to health problems or adverse events [28]. Nurses’ burnout may compromise decision-making, job performance, and patient safety [7]. Nurses who feel overwhelmed tend to be more fatigued and have more difficulties facing job demands or engaging with others [1]. They become cynical, detach from work, and view people—especially patients—like objects [1]. Nurse burnout and less optimal work environment are related to increased staff truancy, decreased job satisfaction, low productivity, and inadequate patient care quality, such as increased pressure injuries, more hospitalizations per 100 residents per year, and lower antipsychotic use [29]. Because this study was conducted in an out-of-normalcy pandemic situation, we highly recommend more research to evaluate the relationships between nurses’ perceptions of nursing care quality, burnout, and telomere length.

### Implications and future research

Nurses are at the forefront of caring for patients with COVID-19 and promoting public health. At the initial wave of the pandemic, nurses had limited time to prepare. They faced severe challenges such as a shortage of personal protective equipment, an influx of high-acuity COVID-19 patients, and piloting new work routines. These factors were significant stressors for nurses.

With the high rate of nurse burnout, this study suggests a critical need to promote nurses’ mental and physical health and well-being. The current literature indicates that it takes strategies at various levels to support nurses’ health, including organizational, unit, and individual support and efforts [30]. Organizational culture and leadership play a significant role in promoting nurses’ work environment and psychological health [31–34]. In addition, nurses’ self-care is crucial [35–37]. However, further studies are necessary to quantify the stressors associated with the COVID-19 pandemic and the long-term effects on stress physiology and molecular age acceleration. This can inform the need for interventions that can help promote health and well-being.

## Limitations

This is a cross-sectional study in which we measured the outcome and exposures in the participants at the same time. Thus, it is challenging to derive causal relationships from this one-time measurement of exposure and outcome. In addition, data collection was interrupted because of the COVID-19 pandemic. This study was initiated before the pandemic, interrupted by the adverse event, and restarted after half a year. This process may have affected nurses’ participation in blood collection, as some did not complete the study. Another limitation is whether nurses’ telomere lengths are shorter than expected for their age, which is beyond the scope of this study.

Furthermore, this study took place in one hospital’s peri-operative units, limiting the findings’ generalization. Lastly, this study included largely perioperative nurses. This population may not have been significantly impacted by COVID demands, and thus any inference about the impact of the pandemic on the nurses is hard to connect. During the COVID-19 pandemic, some elective surgeries had to be placed on hold due to a shortage of resources. Some nurses might be reassigned to work outside of their normal duties or be placed on leave. These out of normalcy situations may have affected the findings of the study. Thus, more research needs to be conducted in this area.

## Conclusions

Nurse burnout is a prevalent phenomenon in healthcare, and this study indicates that nurses currently experience high levels of burnout. Nurses’ cellular biomarker, telomere length, is shorter in the group of nurses during the COVID-19 pandemic than before. Appropriate measures should be implemented to decrease nurses’ burnout symptoms and improve nurses’ psychological and physical health. Nurses, especially those younger than 60, report higher burnout symptoms, particularly emotional exhaustion. This study indicates the need for intervention to promote nurses’ health during the pandemic and beyond. If not appropriately managed, nurse burnout may continue to be a significant issue facing the healthcare system.
